# Using Technologies to Address Caregiving Challenges

**DOI:** 10.1093/geroni/igaa057.3040

**Published:** 2020-12-16

**Authors:** Chaiwoo Lee, John Rudnik, Joseph Coughlin

**Affiliations:** MIT, Cambridge, Massachusetts, United States

## Abstract

As the caregiver ratio declines, technology will play an increasingly important role in supporting formal and informal caregivers. This presentation will report on the particular effects that frontier technologies may have on various tasks associated with caregiving, including assisting with basic Activities of Daily Living (ADLs) and Instrumental Activities of Daily Living (IADLs). The expert panel predicted that different technologies and new products will have varied effects on caregiving tasks, and that some tasks may be more impacted than others. Some of the key opportunities and barriers to integrating technologies into various tasks of caregiving will be discussed.

